# Long-Term Non-invasive Ventilation in Children: Current Use, Indications, and Contraindications

**DOI:** 10.3389/fped.2020.584334

**Published:** 2020-11-05

**Authors:** Jean-Paul Praud

**Affiliations:** Division of Pediatric Pulmonology, University of Sherbrooke, Sherbrooke, QC, Canada

**Keywords:** home ventilation, non-invasive ventilation, neuromuscular disorders, chronic respiratory failure, COVID-19, mouthpiece ventilation

## Abstract

This review focuses on the delivery of non-invasive ventilation—i.e., intermittent positive-pressure ventilation—in children lasting more than 3 months. Several recent reviews have brought to light a dramatic escalation in the use of long-term non-invasive ventilation in children over the last 30 years. This is due both to the growing number of children receiving care for complex and severe diseases necessitating respiratory support and to the availability of LT-NIV equipment that can be used at home. While significant gaps in availability persist for smaller children and especially infants, home LT-NIV for children with chronic respiratory insufficiency has improved their quality of life and decreased the overall cost of care. While long-term NIV is usually delivered during sleep, it can also be delivered 24 h a day in selected patients. Close collaboration between the hospital complex-care team, the home LT-NIV program, and family caregivers is of the utmost importance for successful home LT-NIV. Long-term NIV is indicated for respiratory disorders responsible for chronic alveolar hypoventilation, with the aim to increase life expectancy and maximize quality of life. LT-NIV is considered for conditions that affect respiratory-muscle performance (alterations in central respiratory drive or neuromuscular function) and/or impose an excessive respiratory load (airway obstruction, lung disease, or chest-wall anomalies). Relative contraindications for LT-NIV include the inability of the local medical infrastructure to support home LT-NIV and poor motivation or inability of the patient/caregivers to cooperate or understand recommendations. Anatomic abnormalities that interfere with interface fitting, inability to protect the lower airways due to excessive airway secretions and/or severely impaired swallowing, or failure of LT-NIV to support respiration can lead to considering invasive ventilation via tracheostomy. Of note, providing home LT-NIV during the COVID 19 pandemic has become more challenging. This is due both to the disruption of medical systems and the fear of contaminating care providers and family with aerosols generated by a patient positive for SARS-CoV-2 during NIV. Delay in initiating LT-NIV, decreased frequency of home visits by the home ventilation program, and decreased availability of polysomnography and oximetry/transcutaneous PCO_2_ monitoring are observed. Teleconsultations and telemonitoring are being developed to mitigate these challenges.

## Introduction

Advances in perinatal and pediatric critical care have resulted in an increased number of children of all ages with complex medical conditions, including chronic respiratory insufficiency necessitating long-term respiratory support. The latter includes continuous positive-airway pressure (CPAP) and long-term mechanical ventilation. In addition, the use of high-flow nasal cannulae has been recently reported for long-term respiratory support at home in children not amenable to CPAP or mechanical ventilation (1, 2).

Although long-term mechanical ventilation has been variably defined, it is increasingly considered to be the delivery of mechanical ventilation at least 6 h per day for more than 3 weeks (3). Long-term mechanical ventilation can be delivered invasively (via tracheotomy), or non-invasively. Long-term non-invasive ventilation (LT-NIV) is provided via a nasal, oronasal, or facial mask with a bilevel positive airway pressure machine or a portable ventilator. Negative pressure ventilation, which is still mentioned in rare publications on children with acute respiratory failure (4, 5), is virtually absent from recent reports on home long-term ventilation.

This review specifically focuses on LT-NIV, despite the much wider use of long-term non-invasive CPAP at home in children. It describes the worldwide increase in LT-NIV in recent decades and compares the data from various programs around the globe. This is followed by a review and discussion of the indications and contraindications of LT-NIV.

## Current Worldwide Use of Long-Term Non-Invasive Ventilation

Pediatric long-term ventilation programs, especially at home, have been increasingly reported since the 1980s in high-income countries as well as those facing more challenging socioeconomic conditions. Long-term home ventilation minimizes disruptions to the child's development and family life, while preventing dependence on institutions. It also avoids nosocomial infections, frees beds in hospitals, and reduces healthcare costs. In Australia, the latter have been reported to be 7 times lower at home than on hospital wards and 25 times lower than in intensive-care units (6). Of note, although several studies have described a better quality of life for the patient on long-term mechanical ventilation at home compared to long-term ventilation in the hospital, this is not always the case for the patient's family. Stress related to emotional difficulties, financial problems, isolation, sleep deprivation, and insufficient resources of the home-ventilation program is indeed rather common (7).

### Long-Term Invasive vs. Non-invasive Ventilation for Children at Home

At the same time home-ventilation programs were being developed, an increasing number of children have been put on LT-NIV rather than invasive ventilation in order to prevent tracheostomy-related complications, including acute airway blockade by secretions, accidental decannulation, tracheal injury, and respiratory infections (8). Significant differences in the proportion of children on long-term invasive vs. non-invasive ventilation at home have been reported between countries and centers. The reasons include varying interpretations of the risks of LT-NIV vs. long-term invasive ventilation, the expertise and preferences of the local team, the availability of interfaces for infants and young children, and healthcare costs. Table 1 lists data on national and local long-term mechanical-ventilation programs in children published in English since 2010. One striking difference between programs relates to the proportion of children on invasive vs. non-invasive ventilation. While half of the studies reported <30% of children on invasive ventilation, the proportion ranges from 0% in one UK center (9) to 75% nationally in the UK (10) and even 98% in Taiwan (11), where nearly all children were hospitalized in dedicated respiratory-care wards. In addition, the proportion of children on invasive ventilation most often was largely under 50% in middle-income countries (12–16). Finally, despite a progressive rise in the use of LT-NIV in infants in their first year of life at initiation of home mechanical ventilation, invasive ventilation via tracheostomy often remains a preferred option at that age, especially due to equipment availability and the frequent need for both nighttime and daytime ventilation (17).

**Table 1:** National and local long-term mechanical-ventilation programs in children published in English since 2010.

**Author**	**Country**	**No. of patients**	**Study type**	**Age at initiation**	**Main diagnoses**	**IV/NIV**
Tibbals (4)	Australia (2010)	168	Single-center, retrospective, 1979–2008	Median = 6.2 years (range 0.1–19)	• OSA = 32% • NMD = 25% • Tracheobronchomalacia = 14% • Scoliosis = 12% • CNS = 9%	28/30% CPAP: 37%
Racca et al. (7)	Italy (2011)	362	National, cross-sectional, 2007	Median = 8 years (IQR 4–14)	• Musculoskeletal = 49% • Lung/airway disease = 18% • CNS = 13%	41/59%
Wallis et al. (8)	United Kingdom (2011)	933	National, cross-sectional, 2008	—	• NMD = 43% • Lung/airway disease = 37% • CNS = 18%	75/22% §
Paulides et al. (18)	Netherlands (2012)	197	Single-center, retrospective, 1979–2009	Median = 14.7 years (range 0.5–17.9)	• NMD = 66% • CNS = 17% • Lung/airway disease = 6%	51/49%
Hsia et al. (10)	Taiwan (2012)	139(98% in hospital)	Single-center, retrospective, 1998–2006	Range 0.3–18 years	• CNS = 62% • Lung/airway disease = 13.5% • Genetic anomalies = 11.5% • NMD = 10%	98/2%
Sovtic et al. (19)	Serbia (2012)	29	Single-center, retrospective, 2001–2011	Median = 9.3 years (range 0.5–17.8)	• NMD = 62% • Respiratory/chest/spinal = 38%	40/60%
Amin et al. (11)	Canada (2014)	379	Regional, retrospective, 1991–2011	Median = 9.6 years (IQR: 2.9–13.9)	• Musculoskeletal = 35% • Lung/airway disease = 33% • CNS = 29%	17/83%
Preutthipan et al. (12)	Thailand (2014)	148	Single-center, retrospective, 1995–2012	Mean = 4.6 ± 5.0 years for IV, 9.1 ± 4.4 years for NIV	• OSA = 48% • Lung/airway disease = 17% • NMD = 15%	36/64%
Cancelhina et al. (17)	Portugal (2015)	31	Single-center, retrospective, 1993–2003	Median = 3 years (range 0–13)	• NMD = 39% • Metabolic disease = 23% • CNS = 19%	19/81%
Chatwin et al. (13)	United Kingdom (2015)	449	Single-center, retrospective, 1993–2011	Median = 74 months (range <1–221)	• NMD = 56% • Lung/airway disease = 20% • Congenital Sd = 14%	NIV only
Weiss et al. (20)	Austria (2016)	143	National, cross-sectional, 2013	——	• NMD = 44% • Other neurological = 28% • OSA =8.5% • Chest/spine = 8.5 • Lung = 5%	34/60%CPAP = 6%
Chau et al. (9)	Hong Kong (2017)	96	Single-center, retrospective, 1997–2015	Mean = 5.2 ± 6 years for IV, 10.3 ± 7.6 years for NIV	• NMD = 50% • CNS = 25% • Airways = 18% • Chronic lung disease = 6%	26/56%CPAP: 17%
Nathan et al. (21)	Malaysia (2017)	70	Single-center, retrospective, 2001–2014	Median = 11 months (range 5–22)	• Lower respiratory = 49% • Upper airways = 9% • Chest/spine = 11.5% • NMD = 10% • Cardiac disease = 8.5% • Spinal cord injury = 7%	14/43%CPAP: 43%
Van der Poel et al. (22)	South Africa (2017)	55	Single-center, retrospective, 1994–2015	Median = 3.5 years (range 0.4–17.6)	• NMD = 60% • Various = 18% • CNS = 9% • Chest wall = 3.5% • Spinal = 3.5% • Cardiac = 3.5%	71/29%
Ikeda et al. (14)	Japan (2018)	123	Single-center, retrospective, 2001–2015	Median = 139 months (range 8–223)	• NMD = 36% • Congenital anomaly = 21% • Metabolic/degenerative disease = 20% • Perinatal disorder = 11%	57/43%
Hassani et al. (23)	Iran (2019)	67	Single-center, retrospective, 2013–2015	Mean = 5.2 ± 4.9 years	• Lung/airway disease = 45% • NMD = 31% • Metabolic disease = 13.5% • Others = 10.5%	9/32%CPAP: 49%
Park et al. (24)	Korea (2019)	416	National, cross-sectional, 2016	Median = 6 years (range 2–14)	• NMD = 52% • CNS = 34% • Cardiopulmonary = 14%	49/51%
Leske et al. (15)	Argentina (2020)	244	Single-center, retrospective, 2007–2018	Median = 9.4 years (IQR 3.5–14)	• NMD = 43% • Genetic Sd = 23%	14/86%
Pavone et al. (25)	Italy (2020)	432	Single-center, retrospective, 2000–2017	Median = 6.4 years (IQR 1.2–12.8) for NIV, 2.1 years (IQR 0.8–7.8) for IV	• NMD = 30% • Upper airways = 25% • CNS = 23%	27/73%

*IV, invasive ventilation; NIV, non-invasive ventilation; NMD, neuromuscular disease; CNS, central nervous system disease; CPAP, continuous positive airway pressure; §, one patient with negative pressure ventilation*.

### Scoping Reviews on Long-Term Home Non-invasive Ventilation in Infants and Children

Two recent scoping reviews provided comprehensive data on long-term home non-invasive respiratory support internationally.

Castro-Codesal et al. performed a first scoping review of 289 studies on long-term home non-invasive respiratory support—hence including both LT-NIV and CPAP—in children in 2018. The number of children was not specified. The mean age at initiation of long-term non-invasive respiratory support was 8 ± 3 years. The most frequent conditions were upper-airway disorders and neuromuscular diseases. The reported benefits of long-term non-invasive respiratory support at home included decreases in respiratory symptoms, respiratory exacerbations, and postoperative complications, as well as the avoidance of tracheostomy. In addition, polysomnography showed a decrease in the apnea–hypopnea index as well as improved blood gases and sleep architecture (26).

Bedi et al. conducted a second scoping review on both LT-NIV and CPAP involving 60 studies with 977 infants aged 0–2 years in 2018. Respiratory indications included airway disorders (40%), type I spinal muscular atrophy (23%), congenital central hypoventilation syndrome (10%), and various multiple conditions (27%); chronic lung disease of infancy was not mentioned. The authors concluded that infants with airway disorders seemed to benefit from the treatment due to an improvement in respiratory parameters and the potential for outgrowing part of the problem. The only clear benefit at that age, however, was increased survival for infants with spinal muscular atrophy. Results on many outcomes unfortunately were not available, such as neurodevelopment, facial growth, and quality of life (27).

## Indications for Long-Term Non-invasive Ventilation

### General Considerations

Chronic respiratory failure, defined by the inefficiency of the respiratory system to ensure appropriate blood-gas exchange meeting the metabolic requirements of a subject, can manifest by a decrease in PaO_2_ only, or by both an increased PaCO_2_ and a decreased PaO_2_, indicating chronic alveolar hypoventilation. The former can usually be treated with nasal oxygen therapy alone. In the presence of chronic alveolar hypoventilation, long-term mechanical ventilation might be needed, now usually given in the form of LT-NIV.

Diagnosis of chronic alveolar hypoventilation rests on the identification of chronic hypercapnia. Since, as a rule, alveolar hypoventilation manifests first during sleep, chronic hypercapnia was traditionally identified from an arterial or arterialized capillary blood sample taken upon awakening in the morning. Whole-night recording of transcutaneous PCO_2_ + oximetry has now largely replaced morning blood sampling (28). Conversely to diurnal hypercapnia (PaCO_2_ > 45 mmHg), however, there is no consensual definition of nocturnal hypercapnia. Although the American Academy of Sleep Medicine defines hypercapnia as 25% or more of the recording time spent with a PCO_2_ > 50 mmHg (27), other experts consider this definition overly restrictive (28, 29).

The overall aim of LT-NIV is to increase life expectancy and maximize quality of life by augmenting alveolar ventilation and restoring normocapnia, hence preventing the deleterious consequences of chronic alveolar hypoventilation. This is not always possible, however, for a variety of reasons, such as patient discomfort with LT-NIV, especially with the interface. If normocapnia cannot be achieved, every effort must be made to ensure that home LT-NIV provides for alleviating the clinical symptoms of alveolar hypoventilation (see Table 2), discharging the child earlier from the hospital, and preventing complications such as frequent respiratory infections and failure to thrive.

**Table 2:** Clinical signs of chronic alveolar hypoventilation.

Insomnia, nightmares, and frequent arousals
Nocturnal or early morning headaches
Shortness of breath during activities of daily living
Daytime fatigue, drowsiness and sleepiness, loss of energy
Decrease in intellectual performance
Loss of appetite and weight; impaired swallowing
Recurrent respiratory infections
Signs of cor pulmonale

Today, home mechanical ventilation most often consists in non-invasive positive pressure ventilation during the night, preferentially via a nasal mask. While some patients with excessive non-intentional oral leaks necessitate the use of an oronasal mask for LT-NIV—less often a total face mask—both must be used with great caution due to the risk of lung aspiration during vomiting. This is especially true for infants and small children, as well as for subjects unable to remove their mask because of muscle weakness/paralysis or intellectual disability (30, 31). Nighttime LT-NIV can be insufficient in patients with severe chronic respiratory failure, either permanently or during an acute aggravation (e.g., during respiratory infections). Daytime NIV can then be added to nighttime LT-NIV, either via the mask used at night or via a mouthpiece, depending on the patient's preferences and their capability to use a mouthpiece. The latter has the distinct advantage of allowing the patient to tailor their ventilatory needs to the ventilatory assist and facilitates interactions with relatives. Consequently, it is usually preferred over a nasal mask for daytime ventilation (32).

Delivery of home LT-NIV necessitates the existence of a home ventilation program, whose characteristics have been described (33, 34). Close collaboration is mandatory between the hospital multidisciplinary team (who initiates LT-NIV and supervises the medical follow-up and complex care of the patient), the personnel from the home-ventilation program (who ensures equipment delivery; sets up, monitors, and adjusts LT-NIV as prescribed by the hospital team; and answers emergency calls 24/7), and the family caregivers. Monitoring LT-NIV has become a crucial component in home-ventilation programs. In addition to regular oximetry and transcutaneous PCO_2_ recording, built-in ventilator software allows the monitoring of patient adherence and respiratory variables. Telemonitoring is a growing trend in home-ventilation programs. It is generally well-accepted and viewed as advantageous by both patients and family caregivers, as well as by the personnel from the home ventilation program and the hospital complex-care team. Data on telemonitoring benefits for day-to-day care, mean- to long-term outcome, and cost-effectiveness are, however, very limited, especially in children (33, 35, 36).

### Medical Conditions Treated by Long-Term Non-invasive Ventilation

Chronic respiratory insufficiency can be caused by conditions that affect respiratory-muscle performance (alterations in central respiratory drive or neuromuscular function) and/or impose an excessive respiratory load (airway obstruction, lung disease, or chest-wall restriction) (37, 38). Table 3 lists the potential indications for long-term non-invasive ventilation (39). Examples of alterations in central respiratory drive are congenital central hypoventilation syndrome (40, 41), ROHHAD (rapid-onset obesity, hypothalamic dysfunction, hypoventilation, and autonomic dysregulation) (42, 43) and various brainstem anomalies, such as Chiari type II malformation (44). Cerebral palsy can also lead to central hypoventilation, in addition to upper airway obstruction, spine and chest wall deformity, and/or chronic lung disease (45). Central hypoventilation can also contribute to chronic alveolar hypoventilation in congenital syndromes such as Prader-Willi syndrome (46) or Down syndrome (47). Examples of neuromuscular disorders responsible for chronic respiratory insufficiency in pediatrics include type I and type II spinal muscular atrophy in infants/young children (48), as well as various inherited myopathies, the most common being Duchenne muscular dystrophy in adolescents (49). The use of LT-NIV to manage chronic respiratory insufficiency due to high-level spinal-cord injury has also been reported in a number of patients (50). Congenital or acquired chest-wall deformities, such as severe kyphoscoliosis or spondylothoracic dysplasias, can lead to thoracic insufficiency syndrome and require LT-NIV (49). A number of children with non-surgical, severe upper-airway obstruction insufficiently improved by CPAP can avoid tracheostomy with the use of LT-NIV (27, 51). Chronic lung diseases can also benefit from LT-NIV. These include severe cystic fibrosis in adolescents, in whom LT-NIV has been attempted not only to improve quality of life and prevent deterioration, but also as a bridge to lung transplantation, albeit with mixed evidence (33, 52, 53). LT-NIV has also been utilized for some inherited metabolic disorders, which can variably involve the various components of the respiratory system from the brain to the lungs (54). Conversely, if needed, home ventilation is usually provided via tracheostomy in infants with severe bronchopulmonary dysplasia, most often with pulmonary arterial hypertension (33, 34, 55). And while bronchopulmonary dysplasia is listed as a potential indication, including as a bridge from invasive ventilation to spontaneous ventilation without respiratory support, extensive review of the literature finds very few infants with this diagnosis in reports on home LT-NIV (17, 56, 57). Lastly, morbid obesity associated with upper-airway obstruction, restrictive lung disease, and central hypoventilation (obesity-hypoventilation syndrome) can necessitate LT-NIV (33).

**Table 3:** Potential indications for long-term non-invasive ventilation.

Respiratory pump	Neuromuscular diseases
	Chest wall deformity/kyphoscoliosis
	Spinal cord injury
	Prune-Belly syndrome
Respiratory drive	Congenital central hypoventilation syndrome
	Brain/brainstem injury
	Central nervous system tumors
	Metabolic disorders
Airway	Craniofacial malformations
	Obstructive sleep apnea
	Tracheomalacia
	Bronchomalacia
Pulmonary parenchymal and vascular problems	Chronic lung disease of infancy (bronchopulmonary dysplasia)
	Lung hypoplasia
	Recurrent aspiration syndromes
	Cystic fibrosis
	Congenital heart disease

*With permission from (39)*.

## Contraindications for Long-Term Non-Invasive Ventilation

While it is commonly stated that there are no absolute contraindications for LT-NIV, not all patients are eligible. The following conditions may preclude the initiation or adherence to LT-NIV and may lead to considering alternative treatments, such as invasive ventilation via tracheostomy, in concert with the family and patient. It must however be underlined that most contraindications can be successfully managed through a close collaboration between a hospital-based LT-NIV expert team and the personnel from the home-ventilation program.

### Inability to Protect the Lower Airways and Lack of Cooperation From the Patient and/or Family

Inability to protect the lower airways and lack of cooperation from the patient and/or family are often listed as the most important contraindications.

#### Inability to Protect the Lower Airways

Inability to protect the lower airways, due to bulbar impairment or weakened muscles of the upper aerodigestive tract, can lead to dysphagia and lung aspiration with potentially severe acute, subacute, or chronic consequences. The risk of aspiration can come from sialorrhea or occur with feeding or vomiting. Sialorrhea can be treated with anticholinergic medications or repeated injections of botulinum toxin A into the salivary glands (58). Surgery of the salivary glands is another option, especially in children with cerebral palsy (59). Aspiration during feeding is usually managed first by appropriate texture modification of food, then by enteral feeding via a gastrostomy. Diurnal non-invasive ventilation can also decrease respiratory rate, allowing more time for post-swallow expiratory apnea, consequently decreasing the risk of aspiration (60). Moreover, recent studies have shown that, conversely to nasal ventilation, mouthpiece ventilation during meals represents a significant advantage for swallowing and can allow safe feeding when alternating mouthpiece insufflation and swallowing, especially with non-solid food. Improved swallowing with mouthpiece ventilation is likely due to an increase in lung volumes, as well as a decrease in respiratory rate (61–63).

#### Weak Cough

A weak cough impairing the handling of secretions is usually efficiently managed with assisted-cough techniques, which include manually assisted cough ± inspiratory-air stacking or the use of mechanical in/exsufflation (64).

#### Gastroesophageal Reflux

Gastroesophageal reflux uncontrolled by medications and fundoplication might be considered a contraindication to LT-NIV, especially in children with impaired swallowing. An oronasal mask must not be used in such circumstances (65). The decision to proceed to a trial of LT-NIV with a nasal mask should take into account evidence from human and animal studies showing that positive airway pressure therapy, including NIV, can inhibit gastroesophageal reflux (66, 67).

#### Lack of Cooperation From the Patient

Lack of cooperation from the patient is frequent in infants and young children, as well as in children with intellectual disability. While hindering initiation and adherence to LT-NIV, it is generally less of a problem in experienced centers (34). Problems with interface and headgear fitting—frequently encountered in infants and young children—can thus be rapidly and efficiently overcome. Customized ventilatory parameters, which significantly relieve symptoms, can be found without undue delay. As well, the fear sometimes expressed by older children and adolescents that LT-NIV might aggravate the disease (e.g., by deconditioning the respiratory muscles in Duchenne muscular dystrophy) can be discussed beforehand, and the lack of perception of the benefits of LT-NIV rapidly recognized and dealt with. In all these circumstances, full cooperation from the family is of the utmost importance for successful LT-NIV. A family's lack of cooperation or inability to understand recommendations can preclude or delay LT-NIV.

### Other Relative Contraindications

#### Anatomic Facial Abnormalities

Anatomic facial abnormalities can at times interfere with interface fitting and lead to considering invasive ventilation via tracheostomy (68).

#### Presence of Complications Related to the Interface

The presence of complications related to the interface, such as skin ulcerations or mid-face hypoplasia, should no longer be considered contraindications for continuing LT-NIV. Skin ulcerations should be prevented or cured by alternating several masks with different pressure points or by using a cloth mask (69) or a custom-molded mask (70). Mid-face hypoplasia has been shown to complicate nasal-mask respiratory support in a substantial proportion of children—up to 30% (71)—leading to class III malocclusion (72, 73). Beyond an orthodontic consultation at least yearly, which is recommended for all children with LT-NIV, the use of different masks or the preference for either a cloth mask or a custom-molded mask, might prevent the development of mid-face hypoplasia (74). This, however, remains to be proven.

#### Need for Ventilator Support for More Than 16 h a Day

The need for ventilator support for more than 16 h a day remains a classic indication for invasive ventilation through tracheostomy, especially in small infants. Older patients, who often perceive tracheostomy as a factor decreasing their quality of life, can be ventilated via a nasal mask or a mouthpiece during the day, as a complement to nocturnal nasal-mask ventilation. A mouthpiece is usually the first choice for diurnal ventilation to avoid facial-skin breakdowns and facilitate speaking, eating, swallowing, and coughing (32, 75). Nevertheless, depending on the patient preference or ability to use a mouthpiece, nasal-mask ventilation can also be used around the clock (76, 77). Following reports that diurnal mouthpiece ventilation was associated with longer survival than invasive ventilation (78), many experts now strongly advocate for the use of non-invasive ventilation when both nocturnal and diurnal ventilation are needed.

### Long-Term Non-invasive Ventilation in Children During the COVID-19 Pandemic

The global COVID-19 pandemic caused by SARS-CoV-2 has raised several concerns for patients on LT-NIV. First, children with LT-NIV are clinically vulnerable and at increased risk of respiratory failure during any viral respiratory infection. Hence, they are considered at increased risk for severe COVID-19 infection (79). It still is not clear whether the risk is higher with SARS-CoV-2 than other respiratory viruses, such as influenza viruses (80). Of further importance is the fact that, should the child under LT-NIV have COVID-19, LT-NIV can generate aerosols of viral particles, which represent major risks of contamination for caregivers (81). Guidelines on the care of a patient under LT-NIV at home can be found on a few national society websites. Derived from expert opinions, they include proposals for adapting the ventilator equipment and/or the interface, as well as the advice to deliver LT-NIV in a room isolated from the rest of the house (79, 82–84). This becomes of the utmost importance in cases of suspected or confirmed COVID-19 infection, especially if another vulnerable person is present in the household. In such cases, consideration must be given to delivering LT-NIV away from the home. Delaying the initiation of LT-NIV during the peak of the pandemic is also an option in specific cases. Another aspect of LT-NIV during the COVID-19 pandemic is monitoring home LT-NIV and modifying NIV settings as deemed necessary. Telemonitoring and video consultation are becoming genuine assets in preventing contact between the child's family and the personnel from either the home-ventilation program or the hospital complex-care team. It might be more difficult—even impossible—to obtain a full-night in-hospital polysomnography or even night oximetry + transcutaneous PCO_2_ recording at home to confirm that the LT-NIV settings are adequate. Such lack of usual surveillance might jeopardize the quality of LT-NIV provided to new and/or complex patients.

## Conclusion

Home LT-NIV in children is used increasingly worldwide, including in countries with more challenging socioeconomic conditions. Further developments are foreseen, especially in such countries, and can take advantage, to a certain extent, of programs in place elsewhere. Overall, LT-NIV programs provide marked improvement in terms of decreased mortality and increased quality of life to a number of patients with complex disorders involving chronic respiratory insufficiency. While there are a number of indications for LT-NIV, contraindications are few. Many established and forthcoming national home-ventilation programs must, however, be optimized to allow children from all socioeconomic conditions to get access to LT-NIV when needed. Of further importance is the necessity for NIV equipment companies to engage in better collaboration with clinician experts in pediatric LT-NIV in order to develop further NIV equipment tailored to infants and young children. This includes a greater variety of interfaces (masks and headgears) specifically for infants and children, as well as ventilator algorithms and telemonitoring programs for children of all ages to help monitor the appropriateness of LT-NIV.

## Author Contributions

J-PP is the sole contributor.

## Conflict of Interest

The author declares that the research was conducted in the absence of any commercial or financial relationships that could be construed as a potential conflict of interest.
